# On Hydrocephalus Acutus

**Published:** 1818-10-01

**Authors:** Arch. Blacklock

**Affiliations:** Surgeon. *Dumfries*


### *On HYDROCEPHALUS ACUTUS.*

### By Mr. Archibald Blacklock.

THAT Hydrocephalus has long been ranked among the opprobria of medical science is a melancholy truth, both as it shows the great mortality of the disease, and as it goes far to paralyse the efforts of the physician when called in to combat that formidable enemy. I believe, practitioners in general have little or no reliance on any one remedy, or combination of remedies, that have been from time to time recommended for its cure. My own experience in the treatment of this disease has not been great; yet, I think I have seen a sufficient number of cases to justify an endeavour to call the attention of the profession to the treatment by blood-letting.

The progress of the attack is at first so insidious, and the mischief done often so great, ere the true nature of the malady is developed, that it is but in a limited number of cases that even the depleting plan, which I wish to recommend, can be expected to succeed. But I am convinced, that the greater number of infants and children will recover from hydrocephalus internus, if they are bled *early* and *largely* from the *vessels* of the *head*; and no method seems better adapted for suddenly and effectually depleting the vessels of the brain, than opening the external jugular. To trust to leeches in such cases is, in my opinion, only tampering with a most formidable complaint. The jugular vein may be opened with safety and expedition in the youngest infant; and the quantity of blood extracted can, in this way, be regulated to a nicety. I have in this manner taken in a *full stream*, from the jugular vein of an infant twelve months old, between three and four ounces of blood, after the symptoms of hydrocephalus were so evident as not to be mistaken by any person of even limited experience in the profession; and with immediate and progressive melioration. Every auxiliary means ought to be made use of that can, in the smallest degree, assist the recovery, such as blisters to the head, and the repeated exhibition of strong purgatives. The bowels are in general from the beginning inactive, and not easily moved by the common doses of medicine; the purgatives should, therefore, not be relinquished until the contents of the intestines have been completely evacuated, and even then, they should be repeated at proper intervals. That children treated on this plan will, in many instances, receive a severe shock to their constitutional powers cannot be doubted, but that should not be taken in the balance when life is in such immediate danger; and as we know that much may afterwards be done to renovate the health of the sufferer. The mode of cure which I have here set forth, has been followed by a very eminent physician (Dr. Maxwell) of this town, ever since the year 1794, and with many happy results.

I may here add, that I am perfectly aware, that early bleeding in hydrocephalus was highly recommended by Dr. Rush of Philadelphia, several years ago; but I am mistaken, if it was ever practised decisively (I mean general blood-letting) in this country, except by Dr. Maxwell, and some other practitioners in this place. I would subjoin some cases which occurred to myself, illustrative of the treatment of blood-letting, were it not that such accounts are often supposed to bend towards what is wished to be proved; I consider it therefore as well to state merely the plan of treatment which has been so successful in this quarter, leaving it to the future experience of others to determine, whether or not it is deserving of the recommendation I have given it.

ARCH. BLACKLOCK, Surgeon.

*Dumfries, June* 10, 1818.

